# An Automated, Single Cell Quantitative Imaging Microscopy Approach to Assess Micronucleus Formation, Genotoxicity and Chromosome Instability

**DOI:** 10.3390/cells9020344

**Published:** 2020-02-02

**Authors:** Chloe C. Lepage, Laura L. Thompson, Bradley Larson, Kirk J. McManus

**Affiliations:** 1 Department of Biochemistry & Medical Genetics, University of Manitoba, Winnipeg, MB R3E 0J9, Canada; Chloe.Lepage@umanitoba.ca (C.C.L.); Laura.Thompson@umanitoba.ca (L.L.T.); 2 Research Institute in Oncology and Hematology, CancerCare Manitoba, Winnipeg, MB R3E 0V9, Canada; 3 BioTek Instruments, Inc., Winooski, VT 05404, USA; larsonb@BioTek.com

**Keywords:** micronucleus, micronuclei, genotoxicity, chromosome instability, single cell quantitative imaging microscopy (scQuantIM), cancer

## Abstract

Micronuclei are small, extranuclear bodies that are distinct from the primary cell nucleus. Micronucleus formation is an aberrant event that suggests a history of genotoxic stress or chromosome mis-segregation events. Accordingly, assays evaluating micronucleus formation serve as useful tools within the fields of toxicology and oncology. Here, we describe a novel micronucleus formation assay that utilizes a high-throughput imaging platform and automated image analysis software for accurate detection and rapid quantification of micronuclei at the single cell level. We show that our image analysis parameters are capable of identifying dose-dependent increases in micronucleus formation within three distinct cell lines following treatment with two established genotoxic agents, etoposide or bleomycin. We further show that this assay detects micronuclei induced through silencing of the established chromosome instability gene, *SMC1A*. Thus, the micronucleus formation assay described here is a versatile and efficient alternative to more laborious cytological approaches, and greatly increases throughput, which will be particularly beneficial for large-scale chemical or genetic screens.

## 1. Introduction

Micronuclei are small, membrane bound, DNA-containing bodies located outside the primary cell nucleus and whose presence is synonymous with genome instability. Micronuclei are typically defined as having a diameter ≤ 1/3 the size/area of the primary nucleus [1] and their appearance (i.e., micronucleus formation) is frequently employed as an indicator of an underlying genotoxic stress [2,3]. In the past, many genotoxic studies have sought to determine the impact compounds have on genome stability by manually assessing the presence of micronuclei following a treatment [4,5], while more recent studies have employed micronucleus formation assays to uncover genes with pathogenic implications in cancer [6,7,8]. In this regard, numerous cytological studies have identified micronuclei within various cancer contexts including head and neck, ovarian, and breast cancers [9,10,11]. Interestingly, the presence of micronuclei in precursor lesions is associated with an increased risk of developing certain cancers [12,13,14,15], suggesting that the presence and frequency of micronuclei may hold clinical value as diagnostic and prognostic biomarkers. Thus, the ability to accurately identify and enumerate micronuclei is relevant in both laboratory and clinical settings.

Although micronuclei arise through a variety of mechanisms, they generally manifest from chromosome mis-segregation events stemming from the stress response (reviewed in [16]). In some instances, inadequate or defective DNA double-strand break repair may result in acentric chromosome fragments (i.e., lacking a centromere) that are incapable of forming the requisite kinetochore-microtubule (K-fiber) attachments for proper segregation to occur. Depending when the double-strand break occurs, chromosome congression to the metaphase plate or segregation from the metaphase plate may be adversely impacted, and these acentric fragments fail to incorporate within the primary nucleus and ultimately form micronuclei [17]. In a similar fashion, micronuclei may also arise due to the mis-segregation of whole chromosomes rather than fragments. For example, molecular defects underlying aberrant K-fiber attachments, aberrant microtubule dynamics, defective spindle assembly, and abnormal centrosome biology (e.g., supernumary centrosomes) have all been shown to induce micronucleus formation [18,19,20]. In addition, mitotic slippage can lead to micronucleus clusters, a sub-type of micronuclei, that are often associated with drug resistant cells, genome chaos, and cell survival (reviewed in [16]). In any case, the ultimate fate of a micronucleus is not clear; some may persist within daughter cells, while others may undergo micronuclear rupture [21,22], or re-join the primary nucleus during subsequent mitotic events, leading to a reduction in the number of micronuclei [17]. Accordingly, the appearance and persistence of micronuclei is dynamic and highly dependent on the underlying genome instability events driving their formation.

The underlying molecular events driving micronucleus formation are often associated with chromosome instability (CIN; reviewed in [18,23]), an enabling feature and hallmark of many cancer types [24,25,26]. CIN is defined as an increase in the rate at which whole chromosomes, or large chromosome fragments are gained or lost [25], and is frequently associated with the formation of micronuclei. Despite decades of biochemical, genetic, and clinical studies showing CIN is associated with cellular transformation, intratumoral heterogeneity, metastasis, drug resistance, and poor patient outcomes [18,19,20,27,28], the molecular determinants (i.e., aberrant genes and pathways) underlying micronucleus formation and driving CIN remain poorly understood. Thus, an automated approach capable of accurately quantifying the frequency of micronucleus formation following a diverse array of treatments/conditions (i.e., drugs or genes) in a variety of experimental or clinical contexts will provide the much-needed etiological insight into cancer pathogenesis by identifying the aberrant conditions driving micronucleus formation and CIN.

In this study, we describe the development, optimization, and application of a single cell quantitative imaging microscopy (scQuantIM) approach used to enumerate micronuclei in various experimental and cellular contexts. We first present the critical concepts behind the automated approach, then highlight the key experimental parameters requiring optimization that will enhance the accuracy and reproducibility of the approach. Using two genotoxic compounds and an established CIN gene, we demonstrate the broad-spectrum utility of the scQuantIM approach to rapidly enumerate micronuclei in multiple cellular contexts. We end by highlighting how this approach can be easily adopted to evaluate query compounds (drugs) or candidate genes, in either direct tests or as part of screening libraries. Accordingly, this scQuantIM approach, coupled with the quantitative data it provides, will enable novel studies aimed at determining the severity and impact novel compounds have on genome stability, and will identify novel genes and pathways with implications in CIN and cancer pathogenesis. Although not shown, this automated approach can be easily adjusted and applied in various clinical contexts (e.g., hematological cancers or circulating tumor cells) to assess the induction and prevalence of micronucleus formation, which will ultimately enable novel health outcome analyses aimed at assessing the clinical utility of micronuclei as novel biomarkers of disease. In summary, the ability of the scQuantIM approach to rapidly identify and enumerate micronuclei in various cellular contexts is critical to enable the innovative studies performed and glean unprecedented insight in a diverse array of experimental and clinical settings.

## 2. Materials and Methods

### 2.1. Cell Culture

HCT116 colorectal cancer cells were purchased from American Type Culture Collection (Manassas, VA, USA) and grown in McCoy’s 5A medium (HyClone) supplemented with 10% fetal bovine serum. FT194 and FT246 fallopian tube secretory epithelial cells were generously provided by Dr. R. Drapkin (University of Pennsylvania, USA) and were grown in DMEM/F12 medium (Gibco) supplemented with 2% Ultroser G (Pall Life Sciences, Saint-Germain-en-Laye, France). Cell lines were authenticated based on growth, morphology and spectral karyotyping. All cells were grown at 37 °C in a humidified incubator containing 5% CO_2_.

### 2.2. Dose Response Curves

Dose-dependent changes in micronucleus formation were evaluated by treating cells with the established genotoxic agents, etoposide (Selleck, Houston, TX, USA) and bleomycin (Sigma, Oakville, ON, Canada). Briefly, 1000 HCT116, FT194, or FT246 cells were seeded into 96-well optical bottom plates, permitted to attach for 24 h and subsequently treated in triplicate with a two-fold serial dilution of etoposide (39 nM to 10 μM) or bleomycin (70 nM to 18 μM) and vehicle control (DMSO or H_2_O, respectively). Due to differences in doubling times, cells were permitted to grow for an additional four (HCT116 and FT194) or six (FT246) days, so that equivalent population doublings occurred. Cells were fixed (4% paraformaldehyde) and counterstained (Hoechst 33342, 300 ng/mL) and subjected to scQuantIM as described in the Results.

### 2.3. Gene Silencing

Micronucleus formation was evaluated following the silencing of *SMC1A*, an established CIN gene [29,30]. Briefly, 1000 HCT116, FT194, or FT246 cells were seeded into 96-well optical plates, permitted to attach for 24 h and transfected with a pool of four distinct ON-TARGETplus (Dharmacon) siRNAs targeting SMC1A or Non-targeting control (siControl) with RNAiMax (Invitrogen), as detailed elsewhere [31]. Transfected cells were grown for an additional four (HCT116 and FT194) or six (FT246) days, at which point cells were fixed (paraformaldehyde), counterstained (Hoechst 33342), and subjected to the scQuantIM as described in the Results.

### 2.4. ScQuantIM

To quantify the micronuclei, nine central, non-overlapping images were acquired from each well using a Cytation 3 Cell Imaging Multi-Mode Reader (BioTek) equipped with a 20× objective (0.45 numerical aperture) and a 16-bit gray scale charged couple device camera. Gen5 (BioTek, Winooski, USA) image analysis software (Image Prime and Spot Counting software features) was used to automatically detect and enumerate the total numbers of micronuclei and primary nuclei within each image series from each experimental condition. To optimize micronucleus enumeration, a maximal mean fluorescence intensity filter was applied to each image to eliminate brightly labeled apoptotic or mitotic cells, while a size inclusion filter was employed to limit the analyses to micronuclei with a diameter of ≤1/3 the size of an average (control) nucleus. In this regard, the average nuclear area for each cell line (control conditions) were empirically determined from which the maximum 1/3 area (size) threshold was calculated. Finally, an *x*, *y* image periphery exclusion filter (30 μm) was applied to eliminate partial nuclei located along the image periphery. Figure panels were assembled in Photoshop CS6 (Adobe, San Jose, USA).

### 2.5. Statistical Analyses

To identify changes in micronucleus formation in any given condition (i.e., drug or siRNA) relative to the controls, the mean number of micronuclei/nucleus was calculated by determining the total number of micronuclei/the total number of nuclei ± standard deviation (SD). Dose response curves were generated wherein a dose-dependent mean ± SD was derived from all three technical replicates (at a given dose) that is presented relative to the vehicle control. For gene silencing, the mean frequency of micronuclei for each technical replicate of each experimental or control condition was determined. The fold change in micronucleus formation is presented relative to the siControl and is determined by dividing each technical replicate value by the mean micronucleus frequency of the siControl. Mann–Whitney (MW) tests were performed to determine whether the mean of the ranks between the *SMC1A* silenced and siControl conditions were statistically different, with a *p*-value < 0.05 considered significant. All descriptive statistics (e.g., n, mean, SD), dose response curves, dot plots, and MW tests were generated in Prism v7 (GraphPad, San Diego, USA).

## 3. Results

### 3.1. The Development of a scQuantIM Approach to Determine the Frequency of Micronucleus Formation

The appearance of micronuclei in cells has traditionally been employed as a beacon that signals an underlying genome instability event or the impact of a genotoxic agent or stress [2,3,4]. As genome instability occurs in up to 90% of all cancers [26,32], the formation and prevalence of micronuclei have far-reaching implications for cancer development, progression, and ultimately health outcomes. Unfortunately, however, previous studies involving micronuclei were limited in that they typically only focus on micronucleus detection and rarely determine the frequency of micronuclei within a given experimental or clinical context [2,3,33,34,35]. As a consequence, those studies are inherently limited in their capacity to rank the severity of a specific experimental condition, or to correlate the frequency of micronucleus formation with disease development, progression, and outcomes. Accordingly, there is an unmet need for a quantitative approach capable of rapidly identifying and enumerating micronuclei within a variety of experimental and clinical contexts at the single cell level.

Determining the frequency of micronucleus formation is critical to establish and rank the severity of a specific experimental condition (e.g., drug or gene candidates), and is essential to determine the clinical utility of micronuclei as potential biomarkers of disease progression, treatment response, and patient outcomes. To enable these types of studies mandates the development of an unbiased, reproducible, and automated scQuantIM approach capable of accurately quantifying micronuclei (or the frequency of micronuclei) within a diverse array of experimental and clinical contexts. The central concept of scQuantIM is that the qualitative differences existing between various conditions can be quantified in such a manner that statistical comparisons can be used to identify significant impacts on genome stability. To enable these quantitative comparisons requires all images to be collected in an identical fashion, that is, the image exposure times must be optimized and maintained constant throughout the entire image acquisition process for a given experiment.

**Figure 1:**
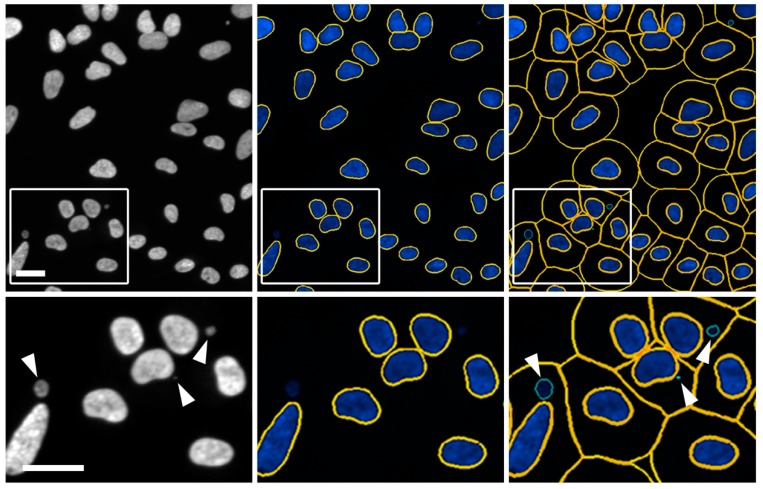
Automated image segmentation and spot detection of micronuclei. Representative images presenting the scQuantIM approach used to detect micronuclei (arrowheads; bottom left image) labelled with Hoechst 33342 in FT246 cells (*SMC1A* silenced conditions). Note that only a portion of a 20× image (i.e., crop image) is presented in the top row, while the bottom row presents the magnified region identified by the bounding box. Gen5 software segments images by applying a primary (nuclear) mask (middle images; yellow lines) and a secondary (cell body) mask (right images; yellow lines), prior to applying spot detection to identify micronuclei (right images; green lines). Scale bar represents 30 μm.

To accurately quantify micronuclei, three key image features (primary nuclei, cell bodies and micronuclei) need to be accurately identified through a process referred to as image segmentation (Figure 1). To segment an image, a primary (nuclear) mask is first applied to define each individual nucleus within a given image, while a secondary (cell body) mask is generated using an average ring width feature (i.e., distance from the nuclear periphery) to approximate the associated cell bodies/boundaries. Finally, spot (micronucleus) detection is used to delineate micronuclei that are spatially located within the secondary (cell body) mask, but are found outside the primary (nuclear) mask. Importantly, each of these key image features must be empirically determined and optimized for each independent cell line employed, and should be established using the control conditions. Note that not all cell lines may be appropriate for this type of analysis. Once segmented, these three key image features can be used to extract quantitative data such as the total number and sizes (areas) of both nuclei and micronuclei. Once extracted, standard statistical analyses can be performed to identify significant differences relative to the controls such as two-sample Kolmogorov-Smirnov (KS) tests comparing the cumulative distribution frequencies of micronuclear sizes (not discussed), or MW tests comparing the rank orders of the mean micronucleus frequencies (detailed below).

### 3.2. Image Segmentation: Key Considerations to Enhance Accurate Data Extraction

To enhance feature recognition and data extraction, several image filters/thresholds should be optimized prior to their consistent application within a given data series. These filters include: (1) A primary (nuclear) mask size filter to ensure only intact nuclei are included in the analyses; (2) a spot (micronucleus) detection size filter set to ≤ 1/3 the size of the average nucleus to ensure only micronuclei are scored; (3) an *x*,*y* image boundary exclusion filter to prevent the inclusion of partial nuclei located along the image periphery; and (4) a Hoechst signal intensity threshold to prevent brightly stained apoptotic or mitotic bodies from being erroneously included in the analyses. The use of additional user-defined inclusion/exclusion criteria may also be critical to ensure accurate image quantification. Size filters including minimum and maximum areas for primary (nuclear) and secondary (cell body) masks, along with spot (micronucleus) detection, can greatly enhance image segmentation and the accuracy of data extraction. For example, the use of a defined ring width for the secondary (cell body) mask will limit the detection of spots (micronuclei) to a defined region surrounding each primary (nuclear) mask. Alternatively, cell boundaries can be readily defined through the use of membrane dyes or the use of antibodies recognizing the cell surface markers; however, these approaches can be time-consuming, costly, and may require optimization prior to experimental execution.

If the image filters/thresholds are not properly optimized prior to data extraction, then a variety of image segmentation and data extraction errors may occur (detailed below). In this regard, many image features may be cell type/line dependent, and thus, will require independent optimization. For example, HCT116 cells generally have smaller nuclei and cell bodies that benefit from smaller mask sizes (particularly for the cell body mask), while FT194 and FT246 typically have larger nuclei and cell bodies that benefit from larger mask sizes. Finally, as CIN is frequently associated with large changes in chromosome complements (e.g., increases in ploidy), the ultimate thresholding parameters employed, especially for the primary (nuclear) mask, must be empirically optimized for each cell line or condition. To assist in this initial optimization step, Table 1 is provided as a reference point as it presents the optimized thresholds and filters employed for HCT116, FT194, and FT246 cells.

### 3.3. Optimizing Image Segmentation: Strategies to Prevent Type I and II Errors

**Figure 2:**
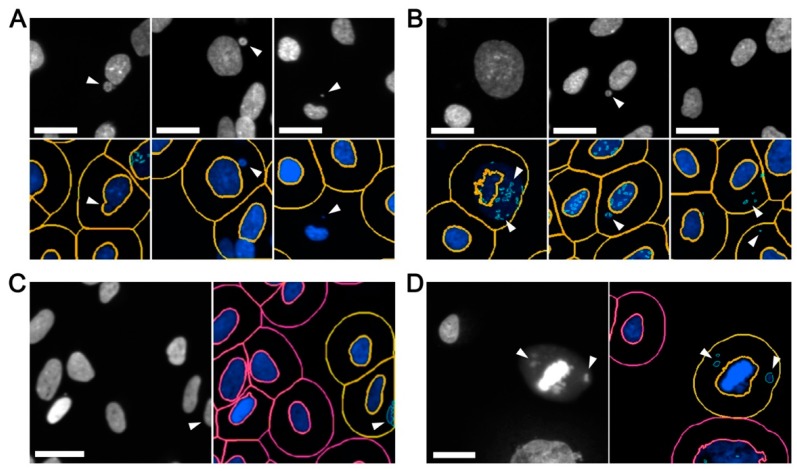
Optimizing image segmentation to enhance micronucleus enumeration. Examples of common detection errors that must be optimized to accurate ensure micronucleus enumeration. Note that only magnified portions of the original 20× images are presented. (**A**) Inadequate image segmentation associated with false negative errors such as micronuclei that are erroneously incorporated into the primary (nuclear) mask (left), micronuclei that are not identified within the secondary (cell body) mask (middle), or, micronuclei located outside the secondary mask (right). Scale bars represent 25 μm. (**B**) Inappropriate image segmentation leading to false positive errors including under segmented images (left and middle), leading to excessive calling of micronuclei, or over segmented images in which background elements are identified as micronuclei (right). Scale bars represent 25 μm. (**C**) Inaccurate segmentation of elements along the image periphery, resulting in false positive errors. Scale bar represents 30 μm. (**D**) Inclusion of mitotic cells in which mitotic chromosomes are erroneously identified as micronuclei. Scale bar represents 30 μm. Note that (A) and (B) are FT246 cells treated with etoposide and bleomycin, respectively, while (C) and (D) are negative controls (dimethyl sulfoxide [DMSO] treated).

The ability to accurately define and detect image features of specific sizes and signal intensities is of paramount importance, as inaccurate image segmentation can lead to both type I (false negative) and type II (false positive) errors. On one hand, false negative errors (Figure 2A) may arise from inaccurate segmentation underlying the inclusion of micronuclei within the primary (nuclear) mask, and/or poor primary mask and spot detection whereby nuclei and micronuclei are not detected. On the other hand, false positive errors (Figure 2B) may arise from: (1) inadequate image segmentation so that only a portion of an intact nucleus is recognized (masked) and the remaining (unmasked) portion is identified as a micronucleus; (2) weak spot detection in which a single micronucleus is recognized as multiple micronuclei; (3) excessive spot detection so that the background (i.e., non-specific) features are identified as micronuclei; (4) image effects in which partial nuclei located along the image periphery are scored as micronuclei (Figure 2C); and (5) apoptotic bodies, mitotic cells, or congressing chromosomes are recognized as nuclei and/or micronuclei (Figure 2D).

Many of the common errors listed above are overcome by adjusting the thresholds and filters employed for a given condition. For example, it is critical to optimize mask (primary and secondary) and spot (micronucleus) detection to enhance feature extraction. Conceptually, inappropriately low thresholds may inadvertently cluster multiple distinct objects (nuclei or micronuclei) into a single object, or fail to recognize small or weakly stained micronuclei. Conversely, overly high thresholds may segment single objects into multiple objects/spots, or erroneously include irrelevant background signals as micronuclei. Beyond mask and spot detection thresholds, image periphery filters should also be applied to prevent partial nuclei from being recognized as micronuclei. In general, restricting the analyses to primary (nuclear) masks located at least 30 μm (*x* and *y* dimensions) from the image periphery (20× image) is highly effective at eliminating these types of errors. Finally, since apoptotic bodies and mitotic chromosomes often fluoresce brighter than interphase nuclei and micronuclei [1], a maximum Hoechst signal intensity threshold should be applied to prevent them from being included in downstream analyses. As a general rule, a maximum intensity threshold should be established by sampling representative regions of images and determining the mean signal intensities of the apoptotic bodies/mitotic cells to be eliminated and the interphase nuclei to be included in the analyses.

To further reduce false negative and positive errors, many image analysis software programs contain additional features that can be employed to enhance image segmentation. For example, in Gen5 (BioTek) these include: (1) A ‘reduce primary mask’ option designed to improve the spatial resolution between the primary nucleus and a proximal micronucleus; (2) a background subtraction option to eliminate non-specific or autofluorescent features that may adversely impact nucleus or micronucleus detection; and (3) a ‘rolling ball’ option to enhance micronucleus detection by better distinguishing non-specific background signals. Finally, Table 2 provides a list of common errors and potential solutions aimed at enhancing image segmentation and feature detection to minimize type I and II errors.

### 3.4. Optimization and Execution of the scQuantIM Workflow

As many common cell lines exhibit genome (karyotypic) instability and/or harbor high levels of micronuclei, it is critical to assess the utility of each cell line prior to evaluating the impact that drug administration or gene silencing may have on micronucleus formation. Accordingly, it is necessary to determine the frequency of micronuclei (on a per cell basis) for each line to be employed in a given study. For demonstration purposes, we carefully selected three different and karyotypically stable cell lines in which to conduct this work: HCT116, FT194, and FT246. Briefly, HCT116 is a malignant colorectal cancer cell line and has been used extensively in CIN-based studies [6,7,29,36], while FT194 and FT246 are non-malignant, hTERT-immortalized fallopian tube secretory epithelial cell lines isolated from two distinct individuals.

To execute the scQuantIM approach, cells (HCT116, FT194, and FT246) are seeded into individual wells of a 96-well plate in triplicate such that they are ~80% confluent on the day of imaging. This ensures that cells are actively growing and are not arrested in G_0_ due to high confluency, which can adversely impact image segmentation. Cells are permitted to attach to the vessel, treated (drugs or siRNAs) and grown for up to 6-days (assay dependent), at which point cells are fixed (paraformaldehyde) and counterstained (Hoechst). Cells are subjected to scQuantIM, where the image exposure time (Hoechst) is first optimized so that the maximum exposure time that produces ~80% of saturated values within typical interphase nuclei is identified. This specific exposure limit allows for dimly stained micronuclei to be more easily quantified, but also accounts for biological variation whereby some nuclei may stain brighter than others.

Once determined, the optimal exposure time is set and maintained at constant throughout the acquisition process. In general, nine non-overlapping images (20×) are collected from the central region of each well to eliminate edge effects like cell stacking along the well periphery. At this magnification, nine images typically provide data on ≥2000 HCT116 primary nuclei from, or ≥1000 FT194/FT246 primary nuclei.

Next, image segmentation is optimized and performed as detailed above and key image feature data are extracted including the total numbers of primary nuclei and micronuclei. To eliminate technical errors and ensure accurate image segmentation (including primary/secondary mask and spot detection), it is imperative to establish the accuracy of the image segmentation through manual (visual) confirmation of a subset of randomly selected images from both the control and experimental conditions. If the image features are not accurately detected/scored, then subsequent rounds of optimization are required prior to data extraction. To account for potential differences in cell confluency resulting from distinct experimental conditions, the frequency of micronuclei in a given well is calculated and expressed as a percentage of the total number of nuclei analyzed within that well. Once collected, all data are imported into a statistical software program (e.g., Prism, GraphPad, San Diego, CA, USA) where statistical tests (e.g., MW tests) are conducted. In general, untreated HCT116, FT194, and FT246 have low frequencies of micronuclei ranging from 1.0% to 3.8% and 2.8%, respectively. These low frequencies (<10%) are consistent with genome (karyotypic) stability and thus identifies each as a suitable cell line model in which to conduct subsequent work.

### 3.5. Determining the Frequency of Micronucleus Formation Following the Administration of Genotoxic Compound

**Figure 3:**
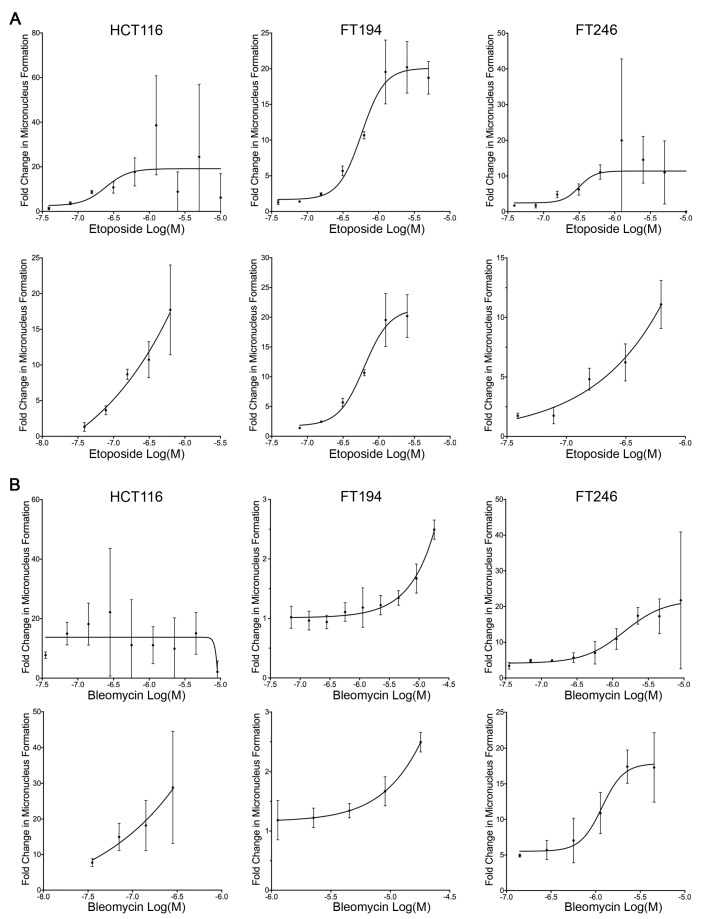
Increasing concentrations of etoposide and bleomycin correspond to increases in micronucleus formation. (**A**) Increasing concentrations of etoposide correspond to increases in micronucleus formation in HCT116 (left), FT194 (middle), and FT246 (right) cells. The (top) graphs present full dose ranges, while the (bottom) graphs present refined ranges. Presented are the mean ± SD for each concentration relative to the vehicle control. Each experimental condition was performed in triplicate. (**B**) Increasing concentrations of bleomycin induce increases in micronucleus formation in HCT116 (left), FT194 (middle), and FT246 (right) cells. (Top) graphs present full dose ranges, while (bottom) graphs present optimal ranges. Presented are the mean ± SD relative to the vehicle control for the experiments performed in triplicate.

To demonstrate the scQuantIM approach, we purposefully selected two well-characterized genotoxic agents, etoposide and bleomycin (reviewed in [37,38]), which both induce micronucleus formation [39,40]. Briefly, etoposide indirectly induces DNA double-strand breaks by inhibiting the re-ligation activity of topoisomerase II [41,42], while bleomycin is a radiomimetic that directly induces single and double-strand breaks via free radical attack [43,44]. To demonstrate the ability of the scQuantIM approach to detect dose-dependent increases in micronucleus formation and establish the limits of detection, cells were treated with a two-fold serial dilution of either etoposide or bleomycin and the vehicle control (see Figure 2 for qualitative examples). Using the experimental approaches detailed above, Figure 3A,B show that increasing concentrations of both etoposide and bleomycin, respectively, induced increases in micronucleus formation within all three lines, with maximum numbers reaching ~20 times those of the vehicle control. Upon further scrutiny, a high degree of variation in the micronuclei counts occurred at higher drug concentrations, which coincides with increases in cell cytotoxicity and an overall decrease in nuclear counts. Accordingly, the increases in well-to-well variation observed at these higher doses simply reflects the large decreases in the cells (nuclei) remaining, as dead and/or dying cells tend to lift off the plates. Further comparisons between these graphs (Figure 3) also renders it possible to gain insights into the innate differences existing between the three different cell lines employed. For example, HCT116 appears to be more sensitive to etoposide and bleomycin (i.e., genotoxic stress) than FT194 and FT246, as HCT116 generally exhibit larger increases in micronuclei at lower doses. In summary, these results establish the utility of the scQuantIM approach in detecting dose-dependent changes in the frequency of micronuclei in various cellular contexts.

### 3.6. Determining the Frequency of Micronucleus Formation Following Gene Silencing

**Figure 4:**
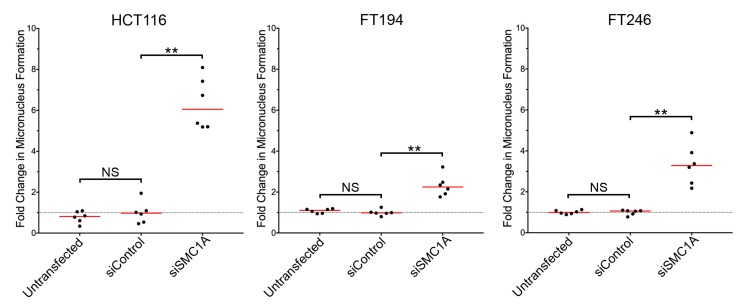
Increases in micronucleus formation accompany reduced *SMC1A* expression. Mann-Whitney tests reveal statistically significant increases in the fold change in micronucleus formation following *SMC1A* silencing relative to siControl in HCT116 (left), FT194 (middle) and FT246 (right) cells (NS, not significant; ** *p*-value < 0.01; N = 3). Data are presented relative to the siControl with red lines identifying the median and the dashed horizontal line identifying the mean of the siControl (1.00).

To establish the ability of the scQuantIM approach to assess genes for their impact on micronucleus formation and CIN, we purposefully selected *SMC1A*, an established CIN gene [29,30]. *SMC1A* encodes an essential member of the sister chromatid cohesion complex, which has established roles in DNA double-strand break repair [45,46] and genome stability [29,30]. Using a similar experimental approach to that detailed above, the frequency of micronucleus formation was determined following *SMC1A* silencing. Briefly, cells were seeded, allowed to attach and grow for 24 h prior to transfection with siRNAs targeting *SMC1A* or a non-targeting control (siControl). Cells were permitted to grow for an additional four (HCT116, FT194) or six (FT246) days, so that equivalent population doublings occurred. Cells were subsequently fixed, counterstained, imaged, and subjected to the scQuantIM analyses, as detailed above (see Figure 1 for qualitative examples). As expected, *SMC1A* silencing corresponded with visual increases in micronucleus formation relative to siControl and untransfected conditions in all three cell lines (Figure 4). In general, *SMC1A* silencing corresponded with a ~3- to 6-fold increase in micronucleus formation that was most pronounced in HCT116 cells and least pronounced within FT194 cells. Subsequent MW tests revealed statistically significant increases in the mean rank of *SMC1A* silenced cells relative to siControl in all three cell lines. These findings are in agreement with those of previous studies, showing that *SMC1A* is a CIN gene in HCT116 cells [29,30], but further establish that *SMC1A* is a CIN gene in FT194 and FT246 cells. In summary, this single example establishes the utility of this scQuantIM approach to quantify micronucleus formation following gene silencing, and thus validates its ability to identify CIN genes.

## 4. Discussion

In this article, we describe the development and application of a scQuantIM approach to detect and analyze micronucleus formation following drug administration or gene silencing. This automated and multi-purpose tool is capable of quantifying micronuclei induced through multiple mechanisms in a variety of cellular contexts including transformed and normal immortalized human cell lines from different tissue types. This scQuantIM approach represents a significant advancement over traditional approaches in that it quantifies the frequency (i.e., level) of micronucleus formation, which may be useful in distinguishing or ranking the level of genotoxic stress ascribed to query compounds or genes. Although the assay was performed using a Cytation 3 Cell Imaging Multi-Mode Reader and Gen5 software (BioTek), the general principles and approaches are readily extractable to virtually any fluorescent microscope (epi-fluorescent or confocal) equipped with image acquisition and analytical software.

Currently, the cytokinesis block micronucleus (CBMN) assay is perhaps the most common approach used for toxicological assessment. In this approach, cytochalasin B (disrupts actin filament formation and cytokinesis) is added to cells to induce the formation of binucleated cells that are subsequently scrutinized for the presence of micronuclei [47]. While the CBMN assay is a traditional endpoint analysis that provides an accurate estimate of micronucleus formation induced following a specific genotoxic exposure, it may not be appropriate in certain contexts. For example, cells harboring genetic defects in CIN genes by definition exhibit persistent and ongoing karyotypic changes, and so the temporal kinetics of micronucleus formation is expected to be dynamic and change with time. Thus, single measurements may not be sufficient to accurately capture the ongoing changes in micronucleus formation within a given population. In addition, the CBMN approach requires multiple labeling steps to delineate nuclei/micronuclei and the cell body, and frequently relies on manual scoring efforts that are subjected to user bias. Automated CBMN algorithms have been devised to identify binucleated cells, but this may not be easily or rapidly achieved on all imaging and analytical systems. Additional in vitro micronucleus tests have also been developed that can be used with or without the use of cytochalasin B [33,35]. In general, these approaches require that cells are exposed to a test chemical and grown to provide sufficient time for chromosomal damage and/or cell cycle proliferation defects to manifest, which are required for micronucleus formation. Many of these approaches involve microscopy, flow cytometry, or image-based flow cytometry and include the use of DNA counterstains (Giemsa or fluorescent DNA dyes), fluorescence in situ hybridization (FISH) probes (chromosome enumeration probes), or kinetochore antibodies to identify micronuclei and/or their contents [48,49,50,51,52,53,54]. As an alternative, our scQuantIM approach does not depend on the efficient inhibition of cytokinesis by cytochalasin B, a compound that by itself has been shown to induce genome instability [34,55] and could conceivably synergize with test compounds or gene silencing to exacerbate micronucleus formation. In contrast, the scQuantIM approach is performed on an asynchronous population of cells. Furthermore, the scQuantIM approach can be easily adapted to live cell imaging through the use of Hoechst 33342 (a membrane permeant DNA counterstain), or through the generation of a cell line model that expresses a fluorescently tagged protein such as histone H2B-green fluorescent protein (H2B-GFP), which will enable live-cell monitoring of the nuclei and micronuclei. Finally, as the scQuantIM approach is automated, it eliminates the user bias/subjectivity commonly associated with manual enumeration to enhance experimental reproducibility and robustness. Overall, the scQuantIM approach presented here offers significant enhancements over traditional approaches and can be easily adapted to a diverse array of conditions including both traditional endpoint and live cell analyses.

The scQuantIM approach is a versatile and rapid tool that will expedite biomedical research. Here, we established the utility of the scQuantIM approach through direct tests (i.e., low throughput) involving both genotoxic agents and a CIN gene. A fundamental limitation of this approach is that it requires the use of adherent cell lines, or that suspension cells be adhered to substrates (detailed below). In addition, the scQuantIM approach is unable to detect micronucleus clusters that generally arise due to mitotic slippage (reviewed in [16]). Although not the principle focus of this report, micronucleus clusters are cytologically distinct and easily identifiable, so they can be manually enumerated within the image series. In any case, this approach is also well-suited to increased throughput and will work equally well in moderate to high throughput screens. In fact, with the appropriate research infrastructure (e.g., automated plate handlers and liquid dispensers), the direct tests presented in this report can easily be expanded into 96-, 384-, or 1536-well plate formats. As such, compound or gene (coding or non-coding [miRNAs, lncRNAs, circRNAs]) libraries can be screened and ranked based on the magnitude of micronucleus formation. Thus, the overall impact reduced gene expression has on micronucleus formation can be assessed using siRNA, shRNA, or CRISPR/Cas9 technologies. In addition to reduced gene expression, overexpression of specific genes is also known to impact genome stability and/or micronucleus formation [56,57,58,59]. Thus, in a similar fashion, direct tests or gene overexpression libraries could also be quantitatively assessed using this scQuantIM approach for their impacts on micronucleus formation and CIN.

A fundamental benefit of the scQuantIM approach is that it can be easily optimized and applied in a variety of contexts, particularly in clinical settings. For example, this approach could be adapted and applied in hematological malignancies where cancer cells are easily isolated. Similarly, circulating tumor cells could be isolated from blood and readily assessed, and they represent a minimally invasive resource that could enable ongoing monitoring of key clinical features including disease progression or treatment response. In both of these circumstances, the suspension cells would need to be adhered to glass microscope slides or within wells prior to imaging, and is readily accomplished using standard approaches including the use of cytospins (centrifugation of cells onto slides), or slides specially coated with poly-l-lysine or fibronectin. Alternatively, the frequency of micronuclei could be determined within solid cancers following the isolation of cancer cells from primary or metastatic sites, or through the interrogation of tissue microarrays housing tumor samples isolated from hundreds of patients. However, this would require additional optimization to account for the 3D nature of nuclei within tissue samples. In any case, the information gleaned from this work will enable novel studies aimed at assessing the clinical utility of micronuclei, or the frequency of micronuclei, as novel disease biomarkers. In this regard, health outcome analyses are easily envisioned that would determine the association between the frequency of micronuclei and disease progression, treatment response, drug resistance, or overall survival. Accordingly, the scQuantIM approach described herein has tremendous implications in cancer, as it will uniquely enable a myriad of future studies in both experimental and clinical settings.

## 5. Conclusions

In summary, we present a scQuantIM approach that accurately detects and determines the frequency of micronuclei within a variety of cellular texts. Importantly, this automated approach represents a significant advancement over traditional manual approaches that are frequently impacted by user bias and fatigue. Through the use of two genotoxic agents and a validated CIN gene, we demonstrated the utility of this approach in quantifying increases in micronucleus formation following treatments. We further highlight that with minimal optimization, this approach is easily scaled to assess compound or gene libraries, and will enable novel studies to identify, rank, and validate those experimental conditions inducing the most severe phenotypes. We also highlight the potential utility of this approach in various clinical settings, highlighting the potential utility of micronuclei as biomarkers of disease. As such, this scQuantIM approach has tremendous potential in a myriad of experimental and clinical contexts and will undoubtedly shed unprecedented insight into the molecular determinants driving disease development, progression, and outcomes.

## Figures and Tables

**Table 1:** Optimized image thresholds and filters employed for the HCT116, FT194, and FT246 cells.

Cell Line	Primary Mask			Secondary Mask	Spot Detection		
	Min OS ^1^ (μm)	Max OS ^2^ (μm)	DT ^3^ (a.u.)	Ring Width (μm)	Min SS ^4^ (μm)	Max SS ^5^ (μm)	DT ^3^ (a.u.)
HCT116	10	100	7000	10	1	5	3000
FT194	10	100	7000	15	1	6	3000
FT246	10	100	7000	15	1	6	3000

^1^ Minimum object size; ^2^ Maximum object size; ^3^ Detection threshold (arbitrary units); ^4^ Minimum spot size; ^5^ Maximum spot size.

**Table 2:** Troubleshooting for common types of spot detection errors.

Error Type	Probable Cause(s)	Interpretation and Potential Solution(s)
Type I (False Negative)	Micronuclei located proximal to a primary (nucleus) mask are not accurately identified and/or segmented	• Primary mask intensity threshold is too low; increase intensity threshold for the primary (nuclear) mask.
		• Distance between the spot (micronucleus) and primary mask (nuclear) is too small; reduce the primary (nuclear) mask size.
	Micronuclei located within a secondary mask are not accurately detected	• Spot (micronucleus) intensity threshold is too high; reduce intensity threshold for spot detection.
		• Spot (micronucleus) detection size is too small; increase maximum spot size.
		• Problem with background flattening parameters; reduce rolling ball size.
	Micronuclei are not included within secondary mask (not detected)	• Poor fitting of primary (nuclear) mask; adjust intensity threshold for the primary (nuclear) mask option.
		• Poor fitting of primary (nuclear) mask; adjust object size of primary (nuclear) mask.
		• Secondary (cell body) mask is too small; increase ring width of secondary (cell body) mask.
Type II (False Positive)	A single object (nucleus or micronucleus) is segmented into multiple objects	• Primary (nuclear) mask intensity threshold is too high; decrease intensity threshold for the primary (nuclear) mask.
		• Spot (micronucleus) detection size is too small; increase maximum spot size.
	Non-specific background labeling is recognized as an object	• Spot (micronucleus) detection intensity threshold is too low; increase intensity threshold for spot detection.
		• Problem with background flattening parameters; increase rolling ball size.
	Objects along the image periphery are erroneously detected/included	• Apply an *x*, *y* exclusion filter in to restrict the analysis to an internal region.
	Mitotic or apoptotic bodies are erroneously included	• Apply a mean object intensity exclusion filter to restrict analysis to primary objects below a particular threshold.
